# Need for speed – finding productive mutations using transcription factor-based biosensors, fluorescenceactivated cell sorting and recombineering

**DOI:** 10.1111/1751-7915.12248

**Published:** 2015-01-28

**Authors:** Michael Bott

**Affiliations:** Institute of Bio- and Geosciences, IBG-1: Biotechnology, Forschungszentrum JülichJülich, D-52428, Germany

The establishment of a bioeconomy requires technologies enabling the rapid development of microbial production strains and enzymes as highly efficient biocatalysts for the conversion of substrates derived from renewable carbon sources into a multitude of chemicals. For strain development, two major strategies are available. The classical approach uses several rounds of random mutagenesis and screening for the desired producer clones and targets the entire genome irrespective of knowledge on function. The rational approach of metabolic and genetic engineering, on the other hand, is based on current knowledge and was strongly enhanced by the development of ‘omics’ technologies and novel tools for genetic engineering, leading to the establishment of systems biology and synthetic biology (Keasling, 2010). Despite the huge success of metabolic engineering, many of the industrial production strains are still based on the classical approach alone or a mixture of both approaches because these strains outperform the ones based exclusively on rational engineering. This is simply due to the fact that our knowledge of even the best studied microorganisms such as *Escherichia coli* or *Saccharomyces cerevisiae* is still far from complete, as obviously shown by the fact that the functions of hundreds of genes in these species are still unknown (because of the lack of a universal strategy to identify these functions). Furthermore, despite the enormous advances in our understanding of enzymes, it is still difficult to predict which mutations in an enzyme will be favourable for a certain purpose. Consequently, random mutagenesis and screening still offer great chances to identify novel mutations that enhance production. Because of the possibility of cheap re-sequencing of clones obtained from screening, the productive mutations can in principle be identified, thus providing the basis for a molecular understanding of the effect of the mutation.

The major bottleneck in the random mutagenesis and screening approach is screening, as the majority of molecules of interest do not have an easily observable phenotype. In the case of amino acid producers, for example, the library of mutagenized clones has to be screened by chromatography or a colorimetric assay. Therefore, the number of variants that can be screened in a reasonable time is much lower than the number of variants that are obtained by random mutagenesis. In the past years, novel tools have been developed that are capable of overcoming the screening bottleneck. The key are genetically encoded biosensors for intracellular molecules. Several types of such sensors have been described, such as those based on Förster resonance energy transfer (Frommer *et al*., 2009) or on RNA switches (Michener and Smolke, 2012). For *in vivo* screening, however, biosensors based on transcriptional regulators (TRs) appear to be the most promising ones, particulary those systems that can be coupled to fluorescence-activated cell sorting (FACS) as an ultrahigh-throughput method allowing screening of more than 50 000 cells per second. A prominent example of such a TR-based biosensor that can be combined with FACS is LysG of *Corynebacterium glutamicum*, a LysR-type TR that activates expression of its target gene *lysE* (encoding a lysine exporter) in response to elevated cytoplasmic levels of L-lysine and a few other amino acids (Bellmann *et al*., 2001). LysG requires binding of L-lysine or L-arginine as inducer in order to activate expression of *lysE* and thus functions as an L-lysine or L-arginine sensor with a *K*_d_ in the low millimolar range. Using a plasmid with a reporter gene coding for an autofluorescent protein like eYFP under the control of the P*_lysE_* promoter, the intracellular lysine concentration can be converted into a fluorescence signal and in a certain concentration range, the cell-specific fluorescence is proportional to the intracellular L-lysine concentration. Importantly, the increased intracellular concentrations correlate with increased extracellular concentrations (Binder *et al*., 2012).

A biosensor like the one based on LysG can be used to screen by FACS libraries generated by random genome mutagenesis, gene-directed mutagenesis or site-directed mutagenesis. For example, using the L-lysine sensor it was possible to screen with FACS in 30 min a *C. glutamicum* library of seven million individual cells obtained by random chemical mutagenesis, a task that would have required weeks or months to do it in a convential way. More than 100 L-lysine-producing cells were isolated from the library, and their analysis led to the identification of novel productive mutations in previously known target genes, but also in novel target genes, such as *murE* (Binder *et al*., 2012). Besides screening of productive mutations in the genome, a targeted approach yielded productive mutations in the key enzymes of L-arginine, L-lysine and L-histidine biosynthesis using the LysG sensor/FACS combination (Schendzielorz *et al*., 2014). For application in site-directed mutagenesis, sensor-based FACS screening can be combined with recombineering, in which site-directed mutations are introduced using single-stranded oligonucleotides and a recombinase. In this way, transformation of a *C. glutamicum* strain carrying a recombinase and the LysG-based sensor with a mixture of 19 oligonucleotides covering all possible amino acid exchanges at *murE*-G81 led to the FACS-based isolation of mutants carrying 12 different productive chromosomal codon exchanges at *murE*-G81 in a single experiment, associated with a spectrum of different lysine titres (Binder *et al*., 2013).

The examples described above demonstrate the power of TR-sensor-based FACS screening for strain and enzyme development, and the number of examples using this combination is increasing rapidly (Mustafi *et al*., 2012; Jha *et al*., 2014; Siedler *et al*., 2014a) and includes also a biosensor for detecting the NADPH/NADP^+^ ratio in *E. coli* cells (Siedler *et al*., 2014b). This type of sensor is suitable for evolving NADPH-dependent dehydrogenases by FACS screening of mutant libraries independent of a specific enzyme assay. Major prerequisites for the use of TR-based FACS screenings are a host microorganism whose morphology is suitable for FACS analysis and the existence of TR-based biosensor for the target molecule. Although thousands of TRs have been identified in the course of genome annotation, for most of them neither their ligand specificity nor their target genes are known. Thus, the question arises how to build a sensor for a molecule for which no TR is known at present. At least two approaches have been reported that address this problem. One is based on altering the ligand specificity of a TR to the molecule of interest. A prominent example is AraC from *E. coli*, whose ligand specificity was changed from L-arabinose to e.g. mevalonate or triacetic acid lactone (Tang and Cirino, 2011; Tang *et al*., 2011). Another approach makes use of metagenomic libraries and is termed SIGEX for substrate-induced gene expression (Uchiyama *et al*., 2005; Uchiyama and Watanabe, 2008). It was developed for the isolation of novel catabolic operons and is based on the fact that such operons are usually induced by the substrate via a TR whose gene is located in close proximity to the catabolic genes. Metagenomic fragments are cloned into an operon trap *gfp*-expression vector, and positive clones becoming fluorescent in the presence of the target molecule are selected by FACS. In this way, TRs for molecules of interest may be identified.

The production of a wide spectrum of chemicals from renewable carbon sources using microbial strains or isolated enzymes requires a shortening of the times required for their development and a maximization of their efficiency. As a complementary approach to metabolic engineering, the use of TR-based biosensors in combination with FACS and next-generation sequencing technologies is a promising route to rapidly identify the key productive mutations, which can then be combined in a suitable host or target protein to obtain superior producer strains or enzymes. Besides their contribution to strain and enzyme development, the recognition of these productive mutations will also increase our understanding of the physiology, metabolism and regulation of cells, and they offer the possibility to discover unexpected linkages in the metabolic network.
